# Coexistence of mastoid, frontal and vertebral hemangiomas in a patient with diabetic neuropathy: Possible correlation between diabetic angiopathy and intraosseous neoangiogenesis

**DOI:** 10.1016/j.radcr.2024.03.087

**Published:** 2024-05-03

**Authors:** Antonio Alessandro Biancardino, Salvatore Marrone, Federica Paolini, Evier Andrea Giovannini, Giovanni Cinquemani, Rita Lipani, Luca Ruggeri, Jaime Mandelli, Antonio Crea, Giuseppe Vaccaro, Domenico Gerardo Iacopino, Luigi Basile

**Affiliations:** a Neurosurgical Clinic, AOUP “Paolo Giaccone”, Post Graduate Residency Program in Neurologic Surgery, Department of Biomedicine Neurosciences and Advanced Diagnostics, School of Medicine, University of Palermo, 90127 Palermo, Italy; b Unit of Neurosurgery, S. Elia Hospital, 93100 Caltanissetta, Italy; c Unit of Diagnostic Imaging, S. Elia Hospital, 93100 Caltanissetta, Italy

**Keywords:** Mastoid hemangiomas, Vascular neoangiogenesis, Diabetes mellitus, Diabetic microangiopathy, Hypoxic-ischemic factors

## Abstract

Bony hemangiomas are benign vascular lesions with an expansive growth; usually they tend to obliterate the entire bony cavity. They are typical lesion of the spinal bones, but they can rarely arise within other bones of the neurocranium. Diabetic microangiopathy is a condition characterized by the development of aberrant vessel tangles anastomosed to each other due to dysregulated neoangiogenesis. We report the case of a 56-year-old woman, suffering from type 2 diabetes mellitus, admitted to the neurology department due to a reported worsening of paresthesias and dysesthesias of the upper and lower limbs. She performed a contrast-enhanced brain CT scan that showed the presence, at the level of the right mastoid process, of an hypervascular angioma. A subsequent MRI study of the brain and spine showed the presence of multiple bone angiomas, at the level of the right frontal theca and C7, Th3, and Th7 vertebral bodies. Due to the absence of further symptoms and clinical and radiological signs of intracranial compression, the patient did not perform surgery. A radiological follow-up was advised. Although possible pathophysiological correlations between diabetes and vertebral hemangiomas are mentioned in literature, vascular lesions of this type involving vertebrae and skull base simultaneously can be discovered in a patient with chronic diabetic disease. As long as these lesions remain asymptomatic, surgical treatment is not indicated, and the patient is followed over time with radiological follow-up.

## Introduction

Intraosseous hemangiomas are benign tumors that account for less than 1% of bone lesions. Although they can arise in different bony districts, vertebral body is a typical location (30%-50%) [1]. According to Mulliken and Glowacki's definition, hemangiomas are histologically characterized by pronounced cell turnover or endothelial hyperplasia, unlike other bone vascular malformations characterized by vessel dysplasia and normal turnover [2]. According to Moore, three histopathological forms are reported in literature: hamartomatous hemangiomas, hemangioblastomas, and sclerosing hemangiomas [3].

They often represent lesion of incidental finding, and they rarely involve different districts outside the cranio-vertebral axis [4]. If located into the temporal bones, these tumors can become osteolytic, infiltrating the tympanic cavity, or can result in exuberant neo-ossification [[5], [6]]. Mastoid hemangiomas are an occasional occurrence that should be investigated, especially if related to the coexistence of other body angiomas [7].

On considering growing characteristics, these lesions often remain stable throughout life, so radiological follow-up is usually the treatment of choice. Surgery is advisable in symptomatic lesions causing nerve compression [8].

The treatment of the first choice is radioembolization with cytoreductive purpose since direct removal may result in significant blood loss [9]. Kostrzewa highlighted the usefulness of CO2 laser treatment in reducing bleeding and hemostatic power [10], while Pavamani applied radiotherapy to very large inoperable lesions [11].

As we know, diabetic pathology is characterized by altered coagulation and abnormalities in myelination of nerve sheath elements causing peripheral neuropathy. Moreover, there seems to be a trend to irregular neoangiogenesis, with the development of aberrant and brittle vessels [12].

Our paper aims to investigate a possible relationship between diabetic neuropathy and the coexistence of multiple bone angiomas. Our hypothesis relates neurovascular abnormalities that are typical of diabetes mellitus with a major trend to angiomas formation.

## Case presentation

A 56-year-old woman with prior history of hypertension and type II diabetes mellitus, diabetic neuropathy in all 4 limbs for about a year and left *a frigore* facial palsy about 30 years ago.

**Fig. 1:**
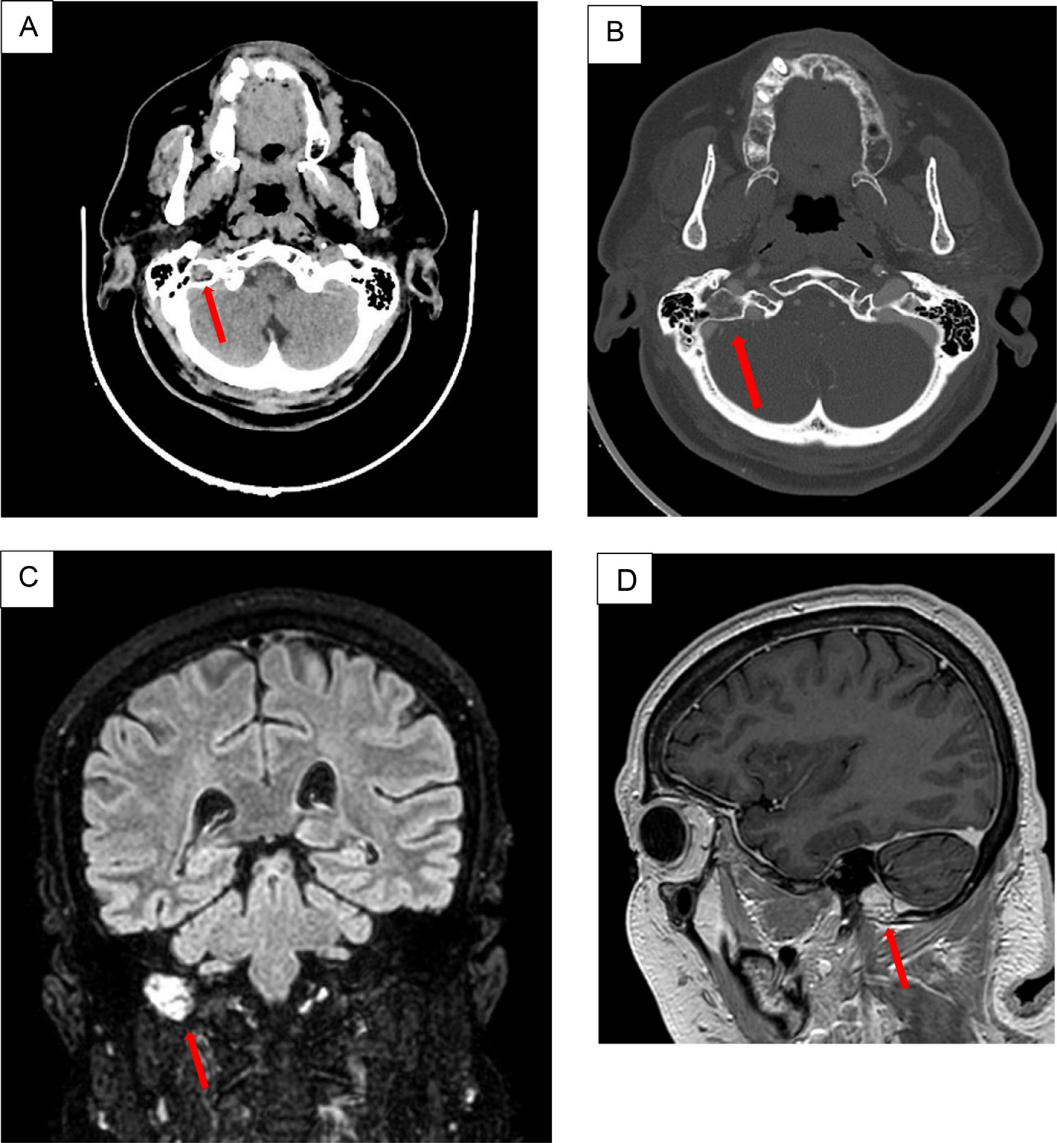
(A) *Axial view*: CT scan shows the presence of oval formation in the medial side of the right mastoid, at occiputmastoid junction, side leading to cancellous bone swelling and bone cortical thinning with parenchymatous-like density. (B) Axial view: CT scan – bone window, slice 1.25 mm**:** note the trabeculation of the bone thinned and remodeled by the presence of the angioma. (C) *Coronal view*: MR Flair shows a hyperintense signal with a “bunch of grapes” appearance in right temporal bone. (D) *Sagittal view*: T1-weighted gadolinium enhanced MR shows expansive.

For worsening neuropathy with paresthesias and dysesthesias in the extremities of hands and feet, she underwent a Contrast Enhanced brain CT study, which documented an oval lesion of 12 × 10 mm in the medial slope of right mastoid, resulting in bony tumefaction and cortical thinning, with parenchymatous density in baseline condition progressively assuming contrast medium, suggestive of hyper vascularized angioma (Fig. 1A and B).

MR study with gadolinium confirmed the presence of an area about 19 × 9 mm in the right mastoid near the occiput-mastoid suture, characterized by T2w hyperintensity, tenuous DWI hypersignal in the absence of ADC restriction, with enhancement suggestive of hypervascularized angioma (Fig. 1C and D).

**Fig. 2:**
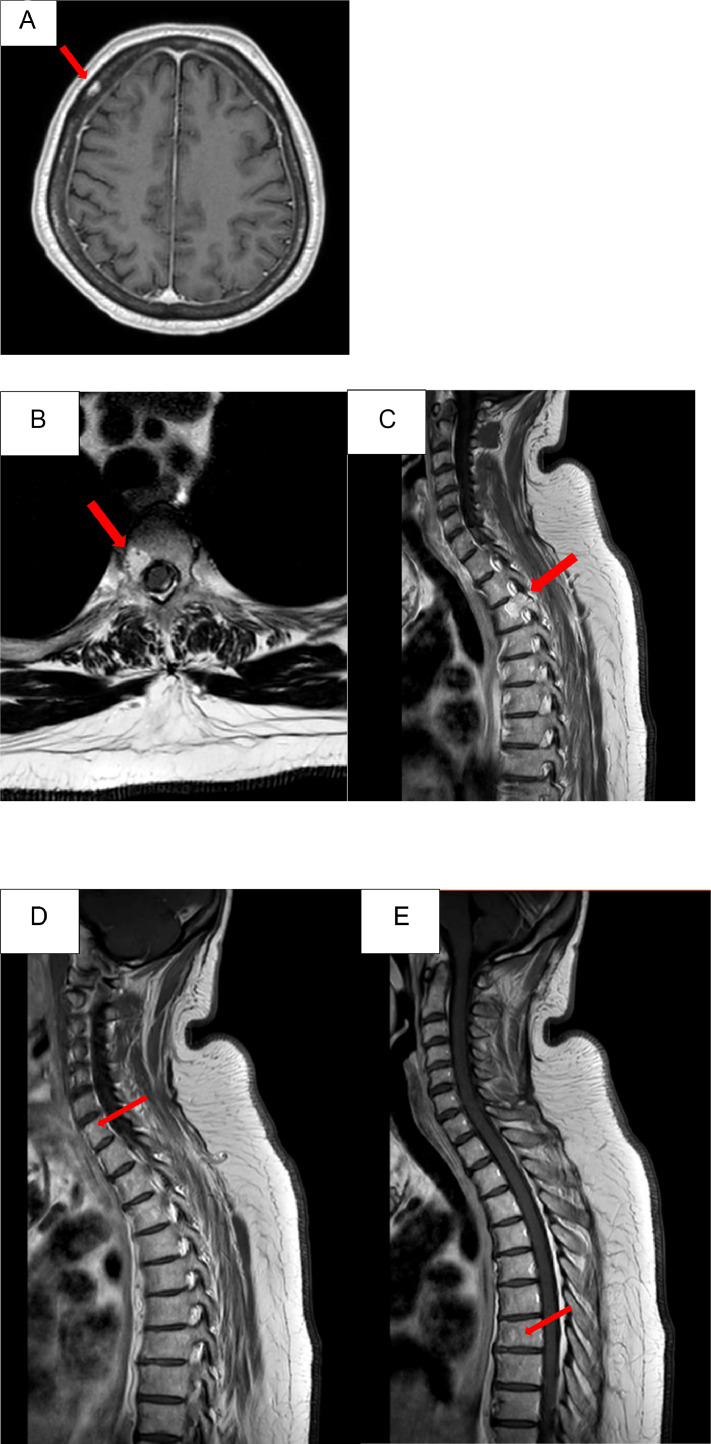
**(A)** Axial view: T1-weighted gadolinium enhanced MR shows small angioma in the right frontal theca. (**B-C)***Sagittal view*: T1-weighted gadolinium enhanced MR and *axial view* T2 MR: show an angioma of the posterior upper aspect of D3 extending to the vertebral peduncle, the transverse process and part of the ipsilateral lamina. **D-E)***Sagittal view*: T1-weighted gadolinium enhanced MR: they show a millimetric angioma at the level of C7 and D7, respectively.

Moreover, several angiomas have been found in different bone location: right frontal theca (Fig. 2A); D3 vertebral body (Fig. 2B and C); C7 and D7 vertebrae (Fig. 2D and E).

Neurological examination was normal, except for paresthesias and dysesthesias. A radiological follow-up was advised.

## Discussion

Bone angiomas are vascular lesions that can affect bony structures of the neuroaxis, usually the vertebral soma, but also cranial bones such as the temporal bones. They are generally benign lesions, highlighted on MRI scans as hyperintense in both T1 and T2 sequences [13]. Rare lesions with malignant character may demonstrate heterogeneous enhancement and irregular margins on MRI scans. CT is mandatory in suspected atypical forms, since it shows a very suggestive polka-dot appearance [14]. The congenital nature of angiomas has been considered in some cases, based on the possibility of the development of acquired vasculopathies in certain systemic and dysmetabolic settings. Cases of hemangiomas developed synovially or cutaneously by repeated mechanical stimulation have been reported in the literature [15].

Among cranial hemangiomas, those in the temporal bone are smaller than the other sites and can sometimes take on an aggressive behavior by invading the middle ear up to cause facial nerve deficits [[6], [16], [17]]. Temporal hemangiomas often originate in areas with dense vascular networks, such as around the geniculate and Scarpa ganglia [[5], [18]]. Diseases such as rheumatoid arthritis, diabetic retinopathy, and tumors such as hemangiomas and psoriasis are driven by uncontrolled angiogenesis [[19], [20]]. Mediators of inflammation and angiogenic growth factors released during trauma or surgical damage could prompt uncontrolled endothelial hyperplasia and microthrombosis. In addition, the same microinstability and locally activated immune response could induce a shift from the normal angiogenetic pathway to an uncontrolled pathological one [21]. Saha and colleagues described Lobular Capillary hemangioma with a focal fibrosis area [22]. Gavilan and colleagues reported ossifying hemangioma case of the temporal bone destructive of normal anatomy by their osteolytic character, with a typical honeycomb appearance on skull CT [23].

When symptomatic, they can be treated by radioembolization or directly surgical exeresis. Treatment aims to prevent the worsening of previous deficits (VII or VIII cranial nerves, usually) [8].

Different treatment modalities are described in literature. Kumbhar and colleagues described a case of temporal petrous hemangioma involving the middle ear and mastoid extending into the retroauricular subcutis, treated by a combination of percutaneous and transarterial embolization [24]. As already ascertained in Slon's study, a correlation between bone angiomas and diabetes mellitus would be plausible [25]. In fact, hyperglycemia and glycation products in chronic diabetes establish a phlogistic microenvironment such that the release of ROS and proangiogenic cytokines is continually urged [[26], [27]]. Moreover, the hypoxic setting due to altered tissue vascularization stimulates HIF activation and the release of neoangiogenesis factors such as PDGF, FGF and VEGF the latter heavily expressed in the dysfunctional endothelium of vertebral angiomas such as in other intraosseous forms [[28], [29]].

## Conclusion

Mastoid hemangiomas are slow-growing benign tumors, among the rarest bony lesions of the cranio-vertebral axis. Generally, they are single lesions but can be part of multiple angiomatosis spectrum with bony angiomas in other body districts. Recent studies in the literature have shown a correlation between the development of bone vasculopathies and several other systemic dysmetabolic diseases. Because of this, even if the exact pathophysiological mechanisms of MHs are still unknown, a correlation between microangiopathy and proliferative neoangiogenesis in diabetic pathology cannot be ruled out.

## Patient consent

Written informed consent for the publication of this report was obtained from the patient.

## References

[1] Bruder E, Perez-AtaydeAR Jundt G, AlomariAI RischewskiJ, Fishman SJ (2009). Vascular lesions of bone in children, adolescents, and young adults. A clinicopathologic reappraisal and application of the ISSVA classification. Virchows Arch.

[2] Mulliken JB, Glowacki J. (1982). Hemangiomas and vascular malformations in infants and children: a classification based on endothelial characteristics. Plast Reconstr Surg.

[3] Laredo JD, Assouline E, Gelbert F, Wybier M, Merland JJ, Tubiana JM. (1990). Vertebral hemangiomas: fat content as a sign of aggressiveness. Radiology.

[4] Abe M, Tabuchi K, Tanaka S, Hodozuka A, Kunishio K, Kubo N, Nishimura Y (2004). Capillary hemangioma of the central nervous system. J Neurosurg.

[5] Watanabe M, Kubo N, Hakozaki S, Kuwata N, Monma N, Ogawa A. (2001). [Intracranial capillary hemangioma: a case report]. No Shinkei Geka.

[6] Ethunandan M. (2024). Management of midfacial and skull vault osseous vascular lesions. Oral Maxillofac Surg Clin North Am.

[7] Mammarella F, Loperfido A, Zelli M, Pianura CM, Bellocchi G. (2020). Middle ear and mastoid diseases: literature review and new classification proposal. Cureus.

[8] Wuyts L, Potvin J, Vanderveken OM, Spaepen M, Lammens M, Van de Heyning P. (2014). Middle ear capillary haemangioma causing vestibulocochlear symptoms: a case report. B-ENT.

[9] Mavrogenis AF, Rossi G, Calabrò T, Altimari G, Rimondi E, Ruggieri P (2012). The role of embolization for hemangiomas. Musculoskelet Surg.

[10] Kostrzewa JP., Bowman MK., Woolley AL. (2010). Middle ear hemangioma: a novel treatment for a rare problem. Int J Pediatr Otorhinolaryngol Extra.

[11] Pavamani SP, Surendrababu NR, Ram TS, Thomas M, Viswanathan PN, Viswanathan FR. (2007). Capillary haemangioma involving the middle and external ear: radiotherapy as a treatment method. Australas Radiol.

[12] Tan S, Zang G, Wang Y, Sun Z, Li Y, Lu C (2021). Differences of angiogenesis factors in tumor and diabetes mellitus. Diabetes Metab Syndr Obes.

[13] George A, Mani V, Noufal A. (2014). Update on the classification of hemangioma. J Oral Maxillofac Pathol.

[14] Hoyle JM, Layfield LJ, Crim J. (2020). The lipid-poor hemangioma: an investigation into the behavior of the “atypical” hemangioma. Skeletal Radiol.

[15] Hernández-Hermoso JA, Moranas-Barrero J, García-Oltra E, Collado-Saenz F, López-Marne S (2021). Location, clinical presentation, diagnostic algorithm and open vs. arthroscopic surgery of knee synovial haemangioma: a report of four cases and a literature review. Front Surg..

[16] Voon Hoong F, Fadzilah I, Iskandar H, Majid A, Goh BS (2013). Capillary haemangioma of external and middle ear. Brunei Int Med J.

[17] Nasi-Kordhishti I, Hempel JM, Ebner FH, Tatagiba M. (2021). Calvarial lesions: overview of imaging features and neurosurgical management. Neurosurg Rev.

[18] Gaudino S, Martucci M, Colantonio R, Lozupone E, Visconti E, Leone A (2015). A systematic approach to vertebral hemangioma. Skeletal Radiol.

[19] Immanuel J, Yun S. (2023). Vascular inflammatory diseases and endothelial phenotypes. Cells.

[20] Wold L, Adler CP (2002) Hemangioma and related lesions. In: Fletcher CDM, Unni KK, Mertens F (Eds.) World health organization classification of tumors pathology and genetics of tumors of soft tissue and bone, vol. 5, pp 321–333

[21] Armaganian G, Adetchessi T, Pech-Gourg G, Blondel B, Dufour H, Fuentes S. (2013). L1 burst fracture with associated vertebral angioma. Orthop Traumatol Surg Res.

[22] Saha J, Sarkar A, Chowdhury S, Debnath T. (2022). Capillary hemangioma of the external auditory canal extending to middle ear and mastoid cavity: a rare case report with review of literature. Indian J Otolaryngol Head Neck Surg.

[23] Gavilán J, Nistal M, Gavilán C, Calvo M. (1990). Ossifying hemangioma of the temporal bone. Arch Otolaryngol Head Neck Surg.

[24] Kumbhar S, Saraf R, Limaye U (2013). Middle ear and mastoid hemangioma treated by neuro-interventional techniques. Indian J. Otol.

[25] Slon V, Peled N, Abbas J, Stein D, Cohen H, Hershkovitz I. (2016). Vertebral hemangiomas and their correlation with other pathologies. Spine (Phila Pa 1976).

[26] Martin A, Komada MR, Sane DC. (2003). Abnormal angiogenesis in diabetes mellitus. Med Res Rev.

[27] Fadini GP, Albiero M, Bonora BM, Avogaro A (2019). Angiogenic abnormalities in diabetes mellitus: mechanistic and clinical aspects. J Clin Endocrinol Metabolism.

[28] Bielenberg DR, Bucana CD, Sanchez R, Mulliken JB, Folkman J, Fidler IJ. (1999). Progressive growth of infantile cutaneous hemangiomas is directly correlated with hyperplasia and angiogenesis of adjacent epidermis and inversely correlated with expression of the endogenous angiogenesis inhibitor, IFN-beta. Int J Oncol.

[29] Takahashi K, Mulliken JB, Kozakewich HP, Rogers RA, Folkman J, Ezekowitz RA. (1994). Cellular markers that distinguish the phases of hemangioma during infancy and childhood. J Clin Invest.

